# The crystal structures of benzyl­ammonium phenyl­acetate and its hydrate

**DOI:** 10.1107/S2056989019000288

**Published:** 2019-01-11

**Authors:** David Hess, Peter Mayer

**Affiliations:** a Institut Laue-Langevin, 71 Avenue des Martyrs, 38000 Grenoble, France; b Ludwig-Maximilians-Universität, Department Chemie, Butenandtstrasse, 5–13, 81377 München, Germany

**Keywords:** crystal structure, hydrogen bonding, Hirshfeld analysis, inter­molecular inter­actions, ammonium carboxyl­ate salts

## Abstract

The title compounds benzyl­ammonium phenyl­acetate, C_7_H_10_N^+^·C_8_H_7_O_2_^−^ (**1**), and its monohydrate, C_7_H_10_N^+^·C_8_H_7_O_2_^−^·H_2_O (**2**), can be obtained by evaporating methano­lic solutions containing equimolar amounts of benzyl­amine and phenyl­acetic acid in the absence and presence of water, respectively. N—H⋯O hydrogen bonds in the crystal structure of **1** lead to the formation of hydro­philic channels running along the *b-*axis direction. The hydrogen-bonding system is best described by fused *R* ^3^_4_(10) ring patterns, often observed in ammonium carboxyl­ate salts. In **2**, the presence of the crystal water leads to the formation of a two-dimensional hydrogen-bonding network. The benzyl moieties in **1** and **2** form hydro­phobic layers in the crystal structures with the aromatic rings adopting edge-to-face arrangements.

## Abstract 2

The crystal packing of benzyl­ammonium phenyl­acetate (**1**) and its hydrate (**2**) is governed by hydrogen bonds formed between the ammonium and acetate groups and the water mol­ecule of crystallization (in **2** only). The benzyl moieties for hydro­phobic layers with the aromatic rings adopting edge-to-face arrangements.

## Chemical context   

Many proteins can self-assemble into insoluble aggregates, so-called amyloids, with a high content of β-strands. Amyloid fibrils are qualitatively similar for different proteins, with filaments of a few nanometers in diameter that can grow up to several micrometers in length (McManus *et al.*, 2016 ▸). The amyloid state of proteins is linked to various human diseases, *e.g*. Alzheimer’s disease (Eisenberg & Jucker, 2012 ▸). Besides proteins, oligopeptides (Ozbas *et al.*, 2004 ▸) down to simple dipeptides (Reches & Gazit, 2003 ▸) and even the amino acid phenyl­alanine (Mossou *et al.*, 2014 ▸; Do *et al.*, 2015 ▸) can also self-assemble into stable nanofilaments in aqueous solution. Apart from the obvious link to amyloid diseases, such structures are also inter­esting for technical applications (Gazit, 2007 ▸; Manna *et al.*, 2015 ▸). Hydrogen bonds between ammonium and carboxyl­ate groups, as well as the presence of hydro­phobic residues (*e.g*. aromatic residues) play an important role in the formation of self-assembled structures of (di)peptides or amino acids (Görbitz, 2010 ▸; Mossou *et al.*, 2014 ▸; Reches & Gazit, 2003 ▸). Similarly, the packing motifs of ammonium carboxyl­ate salts are governed by the formation of hydrogen-bonded networks between the ammonium and carboxyl­ate groups, as well as the nature of the residues of the ammonium and carboxyl­ate residues (Kinbara *et al.*, 1996 ▸; Odendal *et al.*, 2010 ▸).
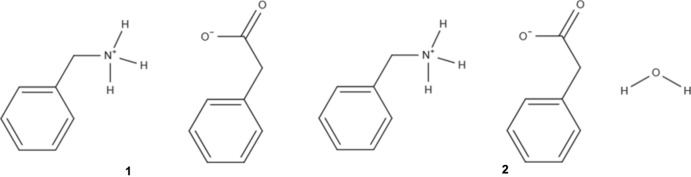


Herein, we report the crystal structures of benzyl­ammonium phenyl­acetate and its hydrate. Both show a similar crystal packing to the zwitterionic form of l-phenyl­alanine reported by Mossou *et al.* (2014 ▸). This resemblance raises the question of whether a system such as benzyl­ammonium phenyl­acetate is also capable of forming nanofilaments.

## Structural commentary   

**Figure 1:**
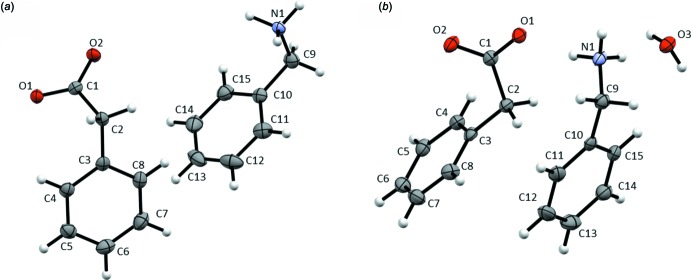
*ORTEP* representation of the asymmetric unit in (*a*) **1** and (*b*) **2** (50% probability ellipsoids).

Benzyl­ammonium phenyl­acetate (**1**) crystallizes in the monoclinic space group *C*2/*c* and its hydrate (**2**) in the monoclinic space group *P*2_1_/*n*. The asymmetric units of **1** and its hydrate **2** are shown in Fig. 1 ▸. In compound **1**, the ammonium group of the benzyl­ammonium is orientated almost perpendicular to the phenyl ring [90.2 (2)°], while the carboxyl­ate group of the phenyl­acetate adopts a torsion angle of −70.2 (4)°, while in the hydrate **2** the torsion angles between the phenyl rings and the functional groups are 72.4 (4) and 54.4 (4)° for the phenyl­acetate and benzyl­ammonium, respectively.

## Supra­molecular features   

### Crystal packing   

**Figure 2:**
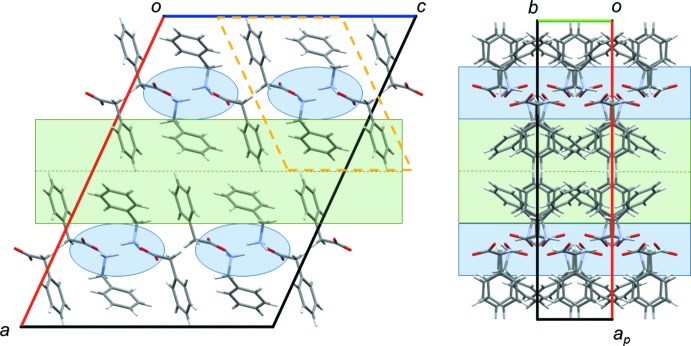
Crystal packing of **1** with views along the *b* axis (left) and along the *c* axis (right). Yellow dotted lines mark a column arranged around a twofold screw axis. Hydro­philic areas are highlighted in blue, hydro­phobic areas in green.

**Figure 3:**
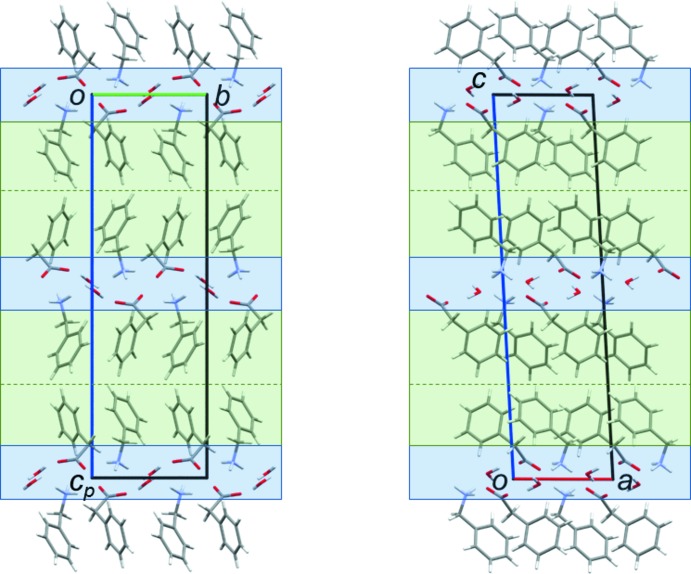
Crystal packing of **2** with views along the *a* axis (left) and along the *b* axis (right). Hydro­philic areas are highlighted in blue, hydro­phobic areas in green.

The crystal packing of benzyl­ammonium phenyl­acetate (**1**) consists of columns arranged around the twofold screw axis along *b* (Fig. 2 ▸). These columns are composed of hydro­philic channels, formed by the ammonium and carboxyl­ate groups, surrounded by a shell made up by the phenyl moieties. The crystal packing of the hydrate (**2**) consists of hydro­philic and hydro­phobic layers alternating along the *c-*axis direction, as shown in Fig. 3 ▸. The hydro­philic layer is composed of the water mol­ecules, the ammonium and the carboxyl­ate groups.

### Inter­molecular contacts and Hirshfeld analysis   

**Figure 4:**
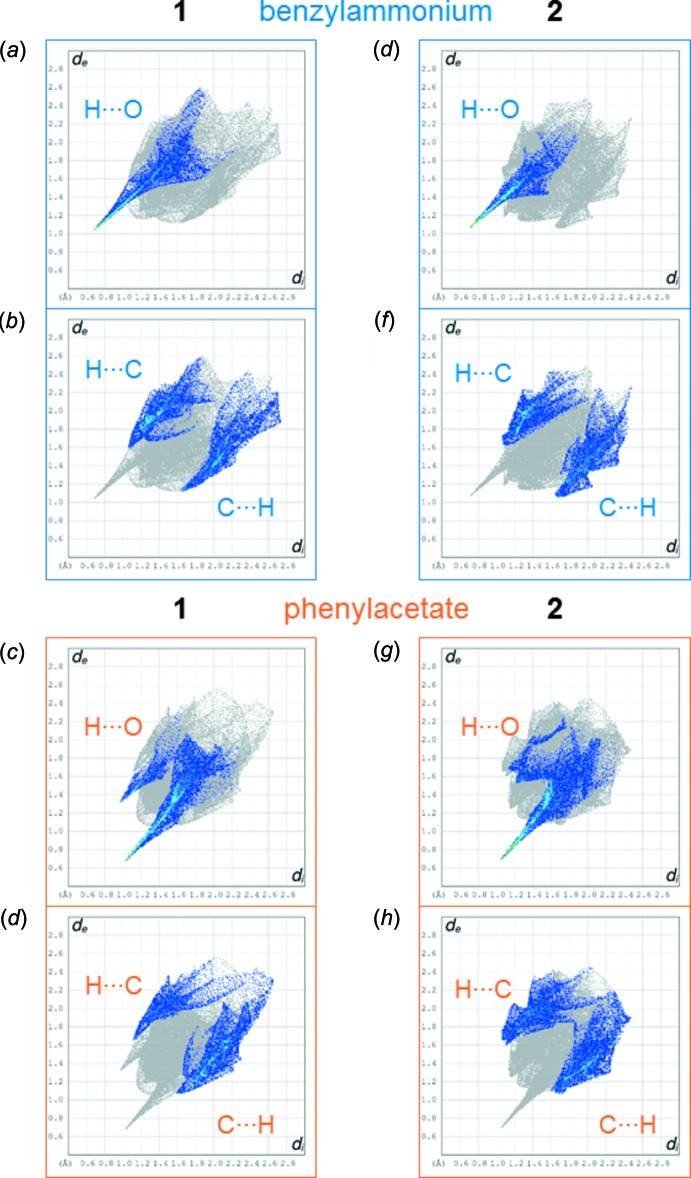
Comparison of the fingerprint plots of the benzyl­ammonium and phenyl­acetate mol­ecules in **1** and **2**, highlighting O⋯H/H⋯O and C⋯H/H⋯C contacts. *d*_i_ and *d*_e_ are plotted in Å on the *x*- and *y*-axis, respectively.

We used *CrystalExplorer17* to analyse the Hirshfeld surfaces of the mol­ecules in the crystal structures of **1** and **2** and to qu­antify inter­molecular contacts between them (Turner *et al.*, 2017 ▸; McKinnon *et al.*, 2007 ▸). Table 1 ▸ summarizes the relative contributions to the Hirshfeld surface areas for the inter­molecular contacts found in the mol­ecules of **1** and **2**. There are three main groups of (inner⋯outer) inter­molecular contacts that can be found on the Hirshfeld surfaces, namely O⋯H/H⋯O, C⋯H/H⋯C and H⋯H inter­molecular contacts. Fig. 4 ▸ shows the fingerprint plots of the benzyl­ammonium and phenyl­acetate mol­ecules in **1** and **2**, highlighting the O⋯H/H⋯O and C⋯H/H⋯C contacts.

**Figure 5:**
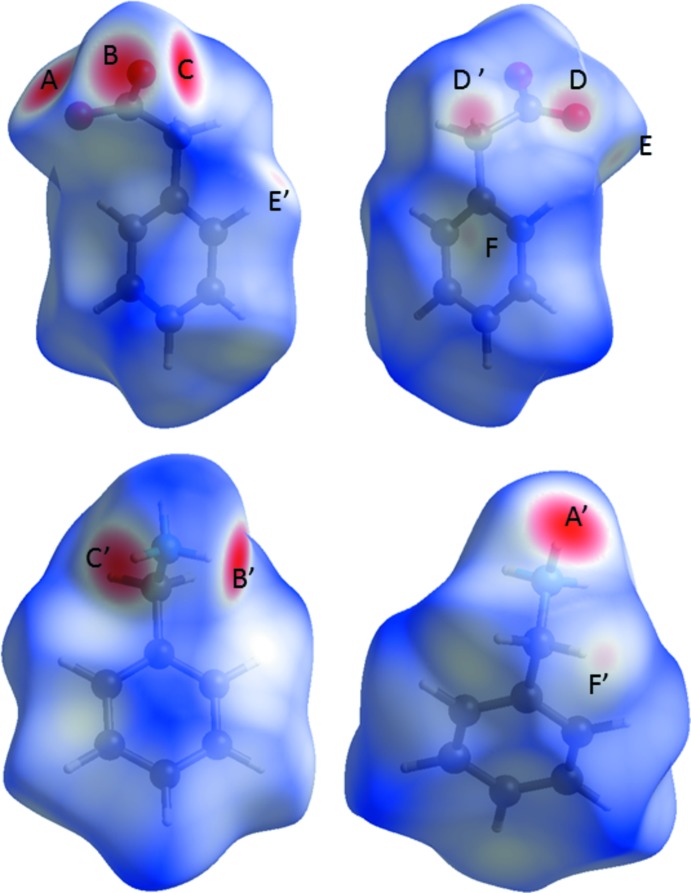
Hirshfeld surfaces of benzyl­ammonium (bottom) and phenyl­acetate (top) mol­ecules in **1** mapped with *d*_norm_. Red spots indicate contact areas shorter than the van der Waals separation. Those contacts can be attributed to the following inter­molecular inter­actions: N1—H11⋯O1 (*A*/*A*′), N—H13⋯O2 (*B*/*B*′), N—H12⋯O2 (*C*/*C*′), C2—H2*B*⋯O1 (*D*/*D*′), C8—H8⋯O1 (*E*/*E*′), and C9—H9*B*⋯π (*F*/*F*′). The map ranges from −0.6825 to 1.3335 a.u. for phenyl­acetate and −0.6822 to 1.4269 a.u. for benzyl­ammonium.

**Figure 6:**
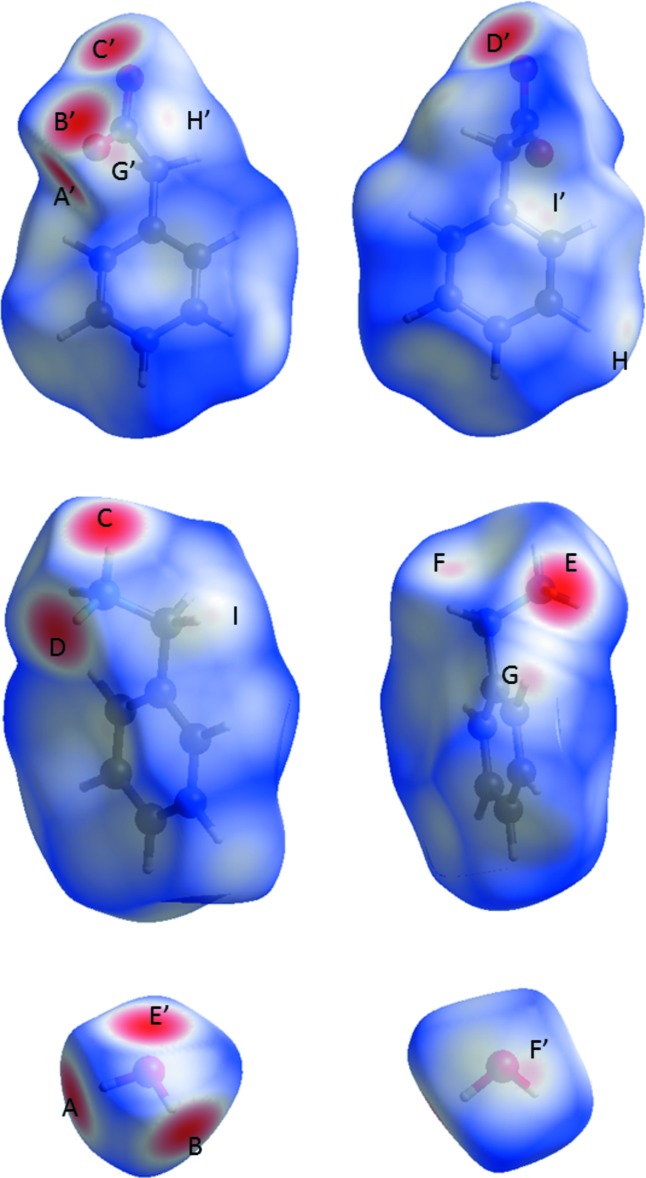
Hirshfeld surfaces of phenyl­acetate (top), benzyl­ammonium (middle) and water (bottom) mol­ecules in **2** mapped with *d*_norm_. Red spots indicate contact areas shorter than the van der Waals separation. Contacts can be attributed to the following inter­molecular inter­actions: O3—H32⋯O2 (*A*/*A*′), O3—H31⋯O2 (*B*/*B*′), N—H12⋯O1 (*C*/*C*′), N—H11⋯O1 (*D*/*D*′), N—H13⋯O3 (*E*/*E*′), C9—H9*B*⋯O3 (*F*/*F*′), C15—H15⋯O2 (*G*/*G*′), C5—H5⋯C1 (*H*/*H*′) and C9—H9*A*⋯C4 (*I*/*I*′). The map ranges from −0.6666 to 1.2024 a.u. for phenyl­acetate, −0.6268 to 1.1600 a.u. for benzyl­ammonium and −0.6680 to 1.0780 a.u. for water.

Mapping the Hirshfeld surfaces with different functions is a helpful tool for visualizing the nature of those inter­molecular contacts. For example, the normalized contact distance *d_norm_* mapped on the Hirshfeld surface using a red–white–blue colour scheme indicates distances shorter, around or greater than the van der Waals separation distances, respectively. The normalized contact distance is defined by the following equation


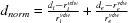
,

where *d_i_* and *d_e_* are the distances to the nearest atoms inside and outside the surface and *r^vdw^* is the van der Waals radius of the appropriate atom inter­nal or external to the surface (McKinnon *et al.*, 2007 ▸). Fig. 5 ▸ shows the benzyl­ammonium and phenyl­acetate mol­ecules in **1** with *d*_norm_ mapped. A number of contacts with distances below the sum of the van der Waals radius can directly be identified by red spots. The most intense ones (*A*/*A*′, *B*/*B*′, *C*/*C*′ in Fig. 5 ▸) can be attributed to N—H⋯O hydrogen bonds between the benzyl­ammonium and phenyl­acetate mol­ecules. The remaining spots are due to non-classical C—H⋯O hydrogen bonds among the phenyl­acetate mol­ecules (*D*/*D*′, *E*/*E*′ in Fig. 5 ▸) and an aliphatic C—H⋯π inter­action between benzyl­ammonium and phenyl­acetate (*F*/*F*′ in Fig. 5 ▸). Fig. 6 ▸ shows the normalized contact distance *d*_norm_ mapped on the Hirshfeld surface of the mol­ecules in **2**, highlighting the N—H⋯O (*C*/*C*′, *D*/*D*′ and *E*/*E*′) and O—H⋯O (*A*/*A*′, *B*/*B*′) hydrogen bonds as the primary inter­molecular inter­actions, followed by the non-classical C—H⋯O hydrogen bonds (*F*/*F*′ and *G*/*G*′). Two further close contacts of the type C—H⋯C (*H*/*H*′ and *I*/*I*′) can be identified.

Fig. 4 ▸ shows the fingerprint plots of the benzyl­ammonium and phenyl­acetate mol­ecules in **1** and **2**. O⋯H/H⋯O contacts can be attributed mainly to classical and non-classical, *i.e.* C—H⋯O, hydrogen bonds. Naturally no O⋯H contacts, but only H⋯O contacts are found on the Hirshfeld surface of the benzyl­ammonium mol­ecules, resulting in a single spike (*i.e*. N—H⋯O hydrogen bonds) highlighted in the fingerprint plots (*a*) and (*e*) in Fig. 4 ▸. The phenyl­acetate mol­ecules can act as hydrogen-bond acceptors *via* their oxygen atoms (*i.e*. O⋯H contacts), visible through the intense spike in the fingerprint plots (*c*) and (*d*) in Fig. 4 ▸. In addition, H⋯O contacts are observed for the phenyl­acetate mol­ecules in **1** and **2**. Such contacts can come from non-classical C—H⋯O hydrogen bonds, where the phenyl­acetate acts as a donor. However, a spike in the fingerprint plots indicating short hydrogen–oxygen distances is only observed for phenyl­acetate in compound **1** (Fig. 4 ▸ *c*) and not in compound **2** (Fig. 4 ▸ *g*), implying that C—H⋯O hydrogen bonds may be more important in **1** than in the hydrate **2**. C⋯H/H⋯C inter­molecular contacts can arise from close ring contacts of the phenyl rings in the hydro­phobic layers, but also from aliphatic C—H⋯π inter­actions. An examination of the crystal packings in Figs. 2 ▸ and 3 ▸ reveals that the phenyl rings are not stacked in a planar, parallel fashion. This is consistent with the absence of C⋯C inter­molecular contacts, which would be expected in such a case (Turner *et al.*, 2017 ▸). O⋯H/H⋯O and C⋯H/H⋯C contacts will be discussed in more detail below.

#### O⋯H/H⋯O inter­molecular contacts   

**Figure 7:**
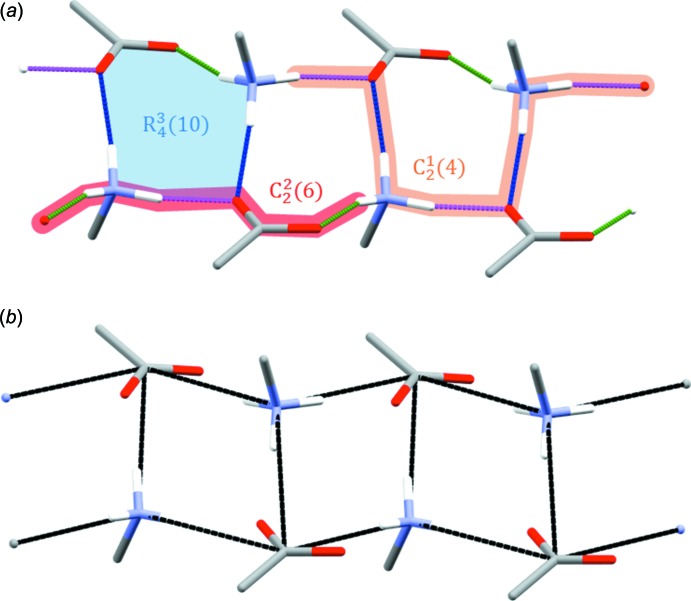
(*a*) Hydrogen-bonding patterns in **1**. A section of the 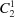
(4) chain pattern is highlighted in orange, and a section of one of the two possible 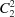
(6) chain patterns is highlighted in red. The 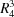
(10) ring pattern is highlighted in blue. Colour code for the hydrogen bonds: N1—H11⋯O1 green, N1—H12⋯O2 magenta, N1—H13⋯O2 blue. (*b*) Cation–anion ladder motif in **1** formed by the repetition of benzyl­ammonium–phenyl­acetate pairs. Phenyl rings and CH_2_ H atoms are omitted for clarity.

As mentioned above, O⋯H/H⋯O contacts can be attributed mainly to classical and non-classical hydrogen bonds. In compound **1**, inter­molecular oxygen–hydrogen contacts amount to about 16 and 26% of the Hirshfeld surface area for the benzyl­ammonium and phenyl­acetate mol­ecules, respectively. In the hydrate **2**, the values are about 13 and 27%, respectively. The hydrogen-bond parameters for **1** and **2** are summarized in Tables 2 ▸ and 3 ▸, respectively. In **1**, the classical hydrogen-bonding system involves the benzyl­ammonium mol­ecule as a donor and the phenyl­acetate mol­ecule as an acceptor for N—H⋯O hydrogen bonds. In **2**, this system is extended by the presence of the water mol­ecule of crystallization acting as a hydrogen-bond donor and acceptor at the same time. The hydrogen-bonding system in **1** can be described by chain patterns corresponding to a second level graph set 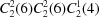 (Bernstein *et al.*, 1995 ▸). However, a more obvious feature is the ring structure denoted by a third level pattern 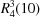 (Fig. 7 ▸ *a*).

**Figure 8:**
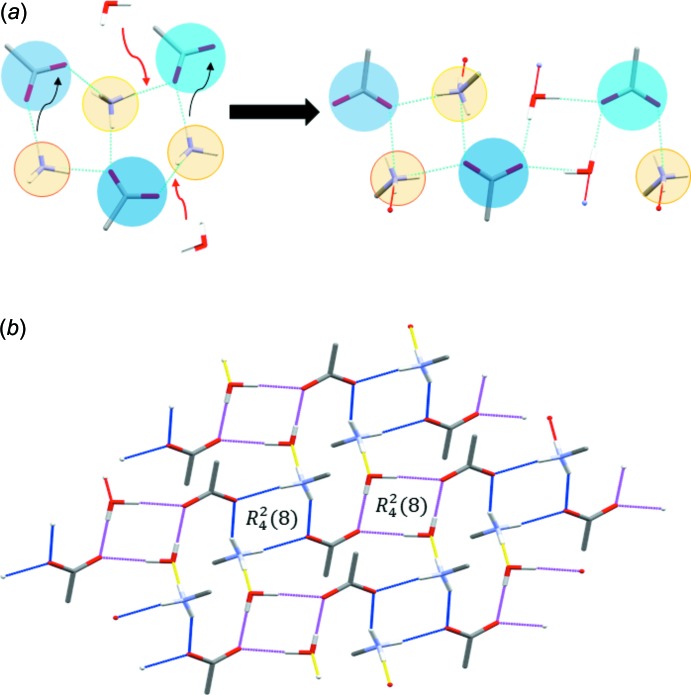
(*a*) Transformation of the hydrogen-bonding network in **1** to the network found in **2** by incorporation of crystal water. (*b*) The two-dimensional hydrogen-bonding network in **2**. Rows of alternating 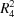
(8) motifs (hydrogen bonds highlighted in blue and magenta, respectively) are connected *via* discrete N—H⋯O hydrogen bonds (highlighted in yellow). Phenyl rings and CH_2_ H atoms are omitted for clarity.

**Figure 9:**
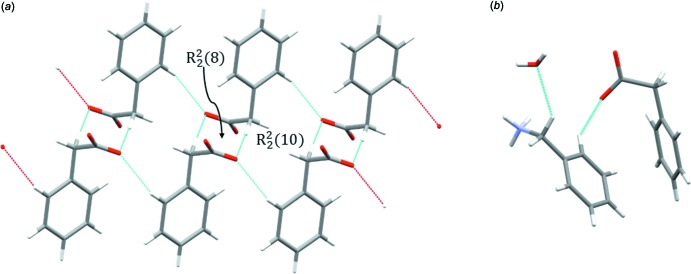
(*a*) The C—H⋯O hydrogen-bonding pattern among the phenyl­acetate mol­ecules in **1** consisting of alternating, fused 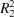
(8) and 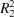
(10)rings. (*b*) Discrete C—H⋯O hydrogen bonds in **2**.

The 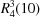 ring pattern is a common feature of ammonium carboxyl­ate salts and has been described earlier (Kinbara *et al.*, 1996 ▸). Related to this particular ring pattern is an electrostatic ladder motif. Two benzyl­ammonium–phenyl­acetate (cation–anion) pairs form a dimeric ring, which associates with further cation–anion pairs to form a ladder running along the twofold screw axis of the crystal (Fig. 7 ▸ *b*). Such a motif is common in ammonium carboxyl­ate salts (Odendal *et al.*, 2010 ▸). Evidently, the presence of crystal water in **2** leads to a change in the hydrogen-bonding system compared to **1**. Going from **1** to **2**, water replaces one of the N—H⋯O bonds between benzyl­ammonium and phenyl­acetate. Consequently, the fused 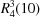 pattern in **1** is disrupted and two alternating 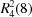 patterns bridged by a carboxyl­ate group are formed (Fig. 8 ▸ *a*). Those rows are then connected among each other *via* the freed N—H donor group of the benzyl­ammonium mol­ecules and water mol­ecules as acceptors to form a two-dimensional hydrogen-bonding network network (Fig. 8 ▸ *b*). Non-classical hydrogen bonds in **1** are formed exclusively between the phenyl­acetate mol­ecules, forming fused 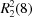 and 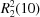 ring patterns alternating along the columns around the twofold screw axis along *b*. The hydrogen-bonding system is shown in Fig. 9 ▸ *a*. In **2**, the benzyl­ammonium mol­ecule acts as a donor for two discrete non-classical C—H⋯O hydrogen bonds (Fig. 9 ▸ *b*), one with the water mol­ecule of crystallization as acceptor (C9—H9*B*⋯O3) and a second one with an oxygen atom of the carboxyl­ate group of phenyl­acetate (C15—H15⋯O2).

#### C⋯H/H⋯C inter­molecular contacts   

**Figure 10:**
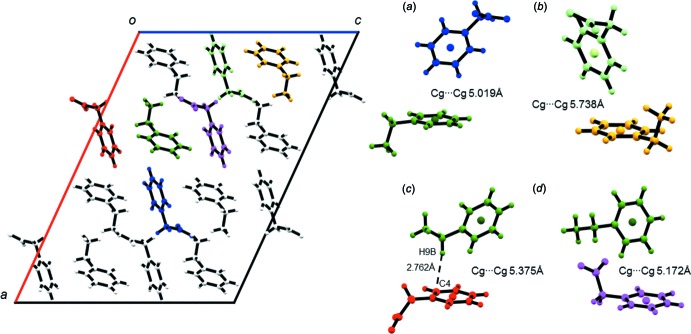
Short ring and aliphatic C—H⋯π inter­actions in **1**.

**Figure 11:**
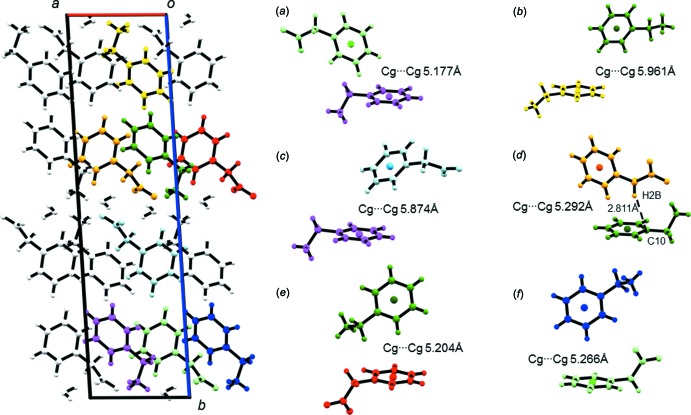
Short ring and aliphatic C—H⋯π inter­actions in **2**.

Carbon–hydrogen inter­molecular contacts contribute to around one quarter of the Hirshfeld surface areas of the benzyl­ammonium and phenyl­acetate mol­ecules in both **1** and **2**. As explained above, those contacts are mainly due to close contacts between the phenyl rings in the hydro­phobic layers of the crystal packing, but also to (aliphatic) C—H⋯π inter­actions. An automated search using *PLATON* (Spek, 2009 ▸) revealed four short ring inter­actions and one aliphatic C—H⋯π inter­action in **1** (Fig. 10 ▸) and six short ring inter­actions and one aliphatic C—H⋯π inter­action in **2** (Fig. 11 ▸). The phenyl rings adopt ‘Y’- and ‘T’-shaped edge-to-face arrangements (Martinez & Iverson, 2012 ▸) with centroid–centroid distances of 5.019 (1)–5.738 (1) Å in **1** and 5.177 (2)–5.961 (2) Å in **2**. Those distances are in the same range as the centroid–centroid distance observed in crystalline benzene (Klebe & Diederich, 1993 ▸). Close H⋯C contacts, *i.e*. smaller than the sum of the van der Waals radii (Bondi, 1964 ▸; Hu *et al.*, 2014 ▸) of the two elements, are found as part of the aliphatic C—H⋯π inter­actions. In **1**, the aliphatic C—H⋯π inter­action is observed between benzyl­ammonium (donor) and phenyl­acetate (acceptor), with the shortest distance being 2.762 Å between C9—H9*B*⋯C4 (Fig. 10 ▸ *c*). In **2**, phenyl­acetate acts as a donor and benzlyammonium as an acceptor for the aliphatic C—H⋯π inter­action. The closest distance of 2.811 Å is found between C2—H2*B*⋯C10 (Fig. 11 ▸ *d*). Two more close contacts of the type (C—)H⋯C can be identified in **2** *via* the *d*_norm_-mapped Hirshfeld surfaces (see *H*/*H*′ and *I*/*I*′ in Fig. 6 ▸). In the first case, the carbon hydrogen distance C5—H5⋯C1 (2.812 Å) between two phenyl­acetate mol­ecules is just below the sum of the van der Waals distances. In the second case, the carbon hydrogen distance C9—H9*A*⋯C4 between benzyl­ammonium and phenyl­acetate is 2.798 Å.

## Database survey   

A structure search on WebCSD (30.11.2018) resulted in 196 hits for structures including benzyl­ammonium and 22 hits for structures including phenyl­acetate. Structures with packings closely related to those of **1** and **2** containing mol­ecules similar to benzyl­ammonium and phenyl­acetate can be found in Trivedi & Dastidar (2006 ▸; CEKJEI, CEKJIM, CEKJOS), Olmstead *et al.* (2008 ▸; HOLDOC), Cai *et al.*. (2009 ▸; BUDQEX), Das *et al.* (2009 ▸; HUKJIH), Mahieux *et al.* (2012 ▸; FAHGIG), Tiritiris & Kantlehner (2011 ▸; HOLDOC01) and Mossou *et al.* (2014 ▸; QQQAUJ03). For a more general view on ammonium carboxyl­ate salts, see Odendal *et al.* (2010 ▸) who described the packing motifs in the crystal structures of such salts, and Kinbara *et al.* (1996 ▸) who described the role of hydrogen-bonded networks in the crystal structures of salts of chiral primary amines with achiral carb­oxy­lic acids.

## Synthesis and crystallization   

Benzyl­amine (185701), phenyl­acetic acid (P16621) and methanol (32213) were obtained from Sigma–Aldrich.

Benzyl­ammonium phenyl­acetate (**1**) was obtained as follows. 40 mg of phenyl­acetic acid (0.29 mmol) were dissolved in 1 ml of methanol and 32 µl of benzyl­amine (0.29 mmol) were added under gentle stirring. The solvent was then evaporated slowly under ambient conditions to yield colourless crystals of compound **1**.

Benzyl­ammonium phenyl­acetate hydrate (**2**) was obtained by dissolving 40 mg of phenyl­acetic acid (0.29 mmol) in 200 µl of methanol and 32 µl of benzyl­amine (0.29 mmol) were added under gentle stirring. The solution was diluted with 1.8 ml of ultra-pure water and evaporated slowly at ambient conditions to yield colourless crystals of compound **2**.

## Refinement   

Crystal data, data collection and structure refinement details are summarized in Table 4 ▸. In **1** and **2**, the C-bound hydrogen atoms were positioned with idealized coordinates (C—H = 0.95–0.99 Å) and refined as riding on their parent atoms with *U*_iso_(H) = 1.2*U*_eq_(N/O). The N-bound hydrogen atoms in **1** were refined freely. In **2**, the coordinates of the N- and O-bound hydrogen atoms were freely refined while the isotropic displacement parameters of the hydrogen atoms were calculated as *U*_iso_(H) = 1.2*U*_eq_(N/O).

## Supplementary Material

Crystal structure: contains datablock(s) 1, 2. DOI: 10.1107/S2056989019000288/vm2215sup1.cif

Structure factors: contains datablock(s) 1. DOI: 10.1107/S2056989019000288/vm22151sup2.hkl

Structure factors: contains datablock(s) 2. DOI: 10.1107/S2056989019000288/vm22152sup3.hkl

CCDC references: 1889253, 1889252

Additional supporting information:  crystallographic information; 3D view; checkCIF report

## Figures and Tables

**Table 1:** Contributions of close inter­molecular contacts to the Hirshfeld surface areas of the mol­ecules in **1** and **2**

Compound	mol­ecule	O⋯H	H⋯O	C⋯H	H⋯C	C⋯O	O⋯C	H⋯H
1	benzyl­ammonium	0.0	15.8	13.6	13.8	1.3	0.0	55.5
	phenyl­acetate	21.5	4.1	16.9	6.6	0.0	0.7	50.2
2	benzyl­ammonium	0.0	13.2	15.6	11.4	0.0	0.0	59.7
	phenyl­acetate	21.6	5.2	15.7	11.3	0.3	0.2	45.7
	water	30.5	22.4	0.0	1.8	0.0	0.0	44.9

**Table 2:** Hydrogen-bond geometry (Å, °) for **1** [Chem scheme1] *Cg*1 is the centroid of the C3–C8 ring.

*D*—H⋯*A*	*D*—H	H⋯*A*	*D*⋯*A*	*D*—H⋯*A*
N1—H11⋯O1^i^	0.97 (2)	1.77 (2)	2.7177 (19)	165 (2)
N1—H12⋯O2^ii^	1.01 (2)	1.73 (2)	2.7306 (19)	170.1 (18)
N1—H13⋯O2^iii^	0.95 (3)	1.85 (3)	2.7938 (19)	174 (2)
C2—H2*B*⋯O1^iv^	0.99	2.40	3.375 (2)	169
C8—H8⋯O1^iii^	0.95	2.63	3.539 (2)	161
C9—H9⋯*Cg*1^v^	0.95	2.92	3.877 (2)	163

Symmetry codes: (i) 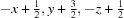; (ii) 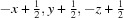; (iii) 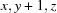; (iv) 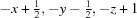; (v) 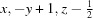.

**Table 3:** Hydrogen-bond geometry (Å, °) for **2** [Chem scheme1]

*D*—H⋯*A*	*D*—H	H⋯*A*	*D*⋯*A*	*D*—H⋯*A*
O3—H32⋯O2^i^	0.83 (4)	1.90 (4)	2.728 (4)	175 (4)
O3—H31⋯O2^ii^	0.97 (4)	1.81 (4)	2.771 (4)	169 (3)
N1—H11⋯O1	0.89 (4)	1.92 (4)	2.791 (4)	168 (4)
N1—H12⋯O1^iii^	0.90 (4)	1.96 (4)	2.805 (4)	157 (4)
N1—H13⋯O3	0.94 (4)	1.87 (4)	2.809 (4)	170 (3)
C9—H9*B*⋯O3^iv^	0.99	2.51	3.196 (4)	126
C15—H15⋯O2^i^	0.95	2.57	3.412 (4)	147

Symmetry codes: (i) 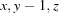; (ii) 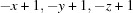; (iii) 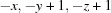; (iv) 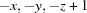.

**Table 4:** Experimental details

	**1**	**2**
Crystal data		
Chemical formula	C_7_H_10_N^+^·C_8_H_7_O_2_^−^	C_7_H_10_N^+^·C_8_H_7_O_2_^−^·H_2_O
*M*_r_	243.29	261.31
Crystal system, space group	Monoclinic, *C*2/*c*	Monoclinic, *P*2_1_/*n*
Temperature (K)	100	100
*a*, *b*, *c* (Å)	25.913 (2), 5.9021 (5), 19.0842 (16)	6.8235 (7), 7.8766 (7), 26.364 (2)
β (°)	114.692 (3)	93.218 (3)
*V* (Å^3^)	2651.9 (4)	1414.7 (2)
*Z*	8	4
Radiation type	Mo *K*α	Mo *K*α
μ (mm^−1^)	0.08	0.09
Crystal size (mm)	0.10 × 0.04 × 0.02	0.10 × 0.06 × 0.04

Data collection		
Diffractometer	Bruker D8 Venture TXS	Bruker D8 Venture TXS
Absorption correction	Multi-scan (*SADABS*; Bruker, 2016 ▸)	Multi-scan (*SADABS*; Bruker, 2016 ▸)
*T*_min_, *T*_max_	0.651, 0.971	0.814, 0.971
No. of measured, independent and observed [*I* > 2σ(*I*)] reflections	14421, 2398, 1800	7319, 2461, 2090
*R*_int_	0.092	0.043
(sin θ/λ)_max_ (Å^−1^)	0.602	0.595

Refinement		
*R*[*F* ^2^ > 2σ(*F* ^2^)], *wR*(*F* ^2^), *S*	0.043, 0.103, 1.03	0.075, 0.170, 1.25
No. of reflections	2398	2461
No. of parameters	175	187
H-atom treatment	H atoms treated by a mixture of independent and constrained refinement	H atoms treated by a mixture of independent and constrained refinement
Δρ_max_, Δρ_min_ (e Å^−3^)	0.17, −0.23	0.30, −0.27

Computer programs: *APEX3* and *SAINT* (Bruker, 2015 ▸), *SHELXT* (Sheldrick, 2015*a* ▸), *SHELXL2014/7* (Sheldrick, 2015*b* ▸), *ORTEP-3 for Windows* (Farrugia, 2012 ▸), *Mercury* (Macrae *et al.*, 2006 ▸), *CrystalExplorer17* (Turner *et al.*, 2017 ▸), *PLATON* (Spek, 2009 ▸) and *RPLUTO* (CCDC, 2018 ▸).

## supplementary crystallographic information

### Benzylammonium phenylacetate (1) Crystal data

**Table:**

C_7_H_10_N^+^·C_8_H_7_O_2_^−^	*F*(000) = 1040
*M**_r_* = 243.29	*D*_x_ = 1.219 Mg m^−^^3^
Monoclinic, *C*2/*c*	Mo *K*α radiation, λ = 0.71073 Å
*a* = 25.913 (2) Å	Cell parameters from 4302 reflections
*b* = 5.9021 (5) Å	θ = 3.5–25.4°
*c* = 19.0842 (16) Å	µ = 0.08 mm^−^^1^
β = 114.692 (3)°	*T* = 100 K
*V* = 2651.9 (4) Å^3^	Block, colourless
*Z* = 8	0.10 × 0.04 × 0.02 mm

### Benzylammonium phenylacetate (1) Data collection

**Table:**

Bruker D8 Venture TXS diffractometer	2398 independent reflections
Radiation source: rotating anode (TXS), Bruker TXS	1800 reflections with *I* > 2σ(*I*)
Focusing mirrors monochromator	*R*_int_ = 0.092
Detector resolution: 7.4074 pixels mm^-1^	θ_max_ = 25.4°, θ_min_ = 3.3°
mix of phi and ω scans	*h* = −30→30
Absorption correction: multi-scan (SADABS; Bruker, 2016)	*k* = −7→7
*T*_min_ = 0.651, *T*_max_ = 0.971	*l* = −22→22
14421 measured reflections	

### Benzylammonium phenylacetate (1) Refinement

**Table:**

Refinement on *F*^2^	0 restraints
Least-squares matrix: full	Hydrogen site location: mixed
*R*[*F*^2^ > 2σ(*F*^2^)] = 0.043	H atoms treated by a mixture of independent and constrained refinement
*wR*(*F*^2^) = 0.103	*w* = 1/[σ^2^(*F*_o_^2^) + (0.0352*P*)^2^ + 1.6577*P*] where *P* = (*F*_o_^2^ + 2*F*_c_^2^)/3
*S* = 1.03	(Δ/σ)_max_ < 0.001
2398 reflections	Δρ_max_ = 0.17 e Å^−^^3^
175 parameters	Δρ_min_ = −0.22 e Å^−^^3^

### Benzylammonium phenylacetate (1) Special details

**Table:**

Geometry. All esds (except the esd in the dihedral angle between two l.s. planes) are estimated using the full covariance matrix. The cell esds are taken into account individually in the estimation of esds in distances, angles and torsion angles; correlations between esds in cell parameters are only used when they are defined by crystal symmetry. An approximate (isotropic) treatment of cell esds is used for estimating esds involving l.s. planes.
Refinement. C-H: constr N-H: refallReflections affected by the beamstop or those of higher order and significant higher Fo^2^ than Fc^2^ (caused by X-ray mirror) have been omitted in the refinement.

### Benzylammonium phenylacetate (1) Fractional atomic coordinates and isotropic or equivalent isotropic displacement parameters (Å^2^)

**Table:**

	*x*	*y*	*z*	*U*_iso_*/*U*_eq_	
O1	0.28279 (5)	−0.35459 (19)	0.41706 (7)	0.0205 (3)	
O2	0.24772 (5)	−0.07061 (19)	0.33362 (7)	0.0214 (3)	
N1	0.27388 (7)	0.8748 (3)	0.20667 (9)	0.0204 (4)	
H11	0.2496 (9)	0.977 (4)	0.1667 (13)	0.040 (6)*	
H12	0.2666 (8)	0.714 (4)	0.1867 (12)	0.035 (6)*	
H13	0.2661 (10)	0.883 (4)	0.2511 (14)	0.048 (7)*	
C1	0.27013 (7)	−0.1506 (3)	0.40169 (10)	0.0169 (4)	
C2	0.28113 (7)	0.0118 (3)	0.46855 (10)	0.0190 (4)	
H2A	0.2687	0.1656	0.4476	0.023*	
H2B	0.2577	−0.0353	0.4958	0.023*	
C3	0.34259 (7)	0.0220 (3)	0.52591 (10)	0.0186 (4)	
C4	0.36866 (8)	−0.1595 (3)	0.57485 (10)	0.0223 (4)	
H4	0.3476	−0.2937	0.5719	0.027*	
C5	0.42493 (8)	−0.1460 (3)	0.62768 (11)	0.0266 (4)	
H5	0.4421	−0.2705	0.6608	0.032*	
C6	0.45623 (8)	0.0480 (3)	0.63248 (11)	0.0307 (5)	
H6	0.4948	0.0573	0.6688	0.037*	
C7	0.43098 (8)	0.2279 (3)	0.58411 (12)	0.0321 (5)	
H7	0.4523	0.3611	0.5869	0.038*	
C8	0.37457 (8)	0.2153 (3)	0.53139 (11)	0.0255 (4)	
H8	0.3576	0.3407	0.4986	0.031*	
C9	0.33454 (8)	0.9379 (3)	0.22888 (11)	0.0273 (5)	
H9A	0.3411	1.0948	0.2491	0.033*	
H9B	0.3428	0.9326	0.1828	0.033*	
C10	0.37371 (7)	0.7794 (3)	0.28918 (10)	0.0220 (4)	
C11	0.38860 (8)	0.8179 (3)	0.36684 (11)	0.0281 (5)	
H11A	0.3763	0.9525	0.3826	0.034*	
C12	0.42124 (8)	0.6622 (4)	0.42177 (11)	0.0354 (5)	
H12A	0.4310	0.6898	0.4748	0.042*	
C13	0.43957 (9)	0.4672 (4)	0.39950 (13)	0.0375 (5)	
H13A	0.4614	0.3592	0.4371	0.045*	
C14	0.42609 (8)	0.4292 (3)	0.32244 (13)	0.0357 (5)	
H14	0.4394	0.2962	0.3072	0.043*	
C15	0.39330 (8)	0.5841 (3)	0.26732 (12)	0.0296 (5)	
H15	0.3841	0.5570	0.2144	0.035*	

### Benzylammonium phenylacetate (1) Atomic displacement parameters (Å^2^)

**Table:**

	*U*^11^	*U*^22^	*U*^33^	*U*^12^	*U*^13^	*U*^23^
O1	0.0266 (7)	0.0168 (7)	0.0177 (6)	0.0002 (5)	0.0089 (5)	0.0000 (5)
O2	0.0305 (7)	0.0202 (7)	0.0134 (6)	0.0023 (5)	0.0091 (5)	0.0006 (5)
N1	0.0279 (9)	0.0181 (9)	0.0150 (8)	0.0000 (7)	0.0089 (7)	0.0013 (7)
C1	0.0161 (9)	0.0203 (10)	0.0168 (9)	−0.0024 (7)	0.0093 (7)	−0.0017 (7)
C2	0.0233 (10)	0.0179 (9)	0.0177 (9)	0.0015 (7)	0.0104 (8)	0.0006 (7)
C3	0.0246 (10)	0.0200 (9)	0.0152 (9)	0.0000 (7)	0.0122 (8)	−0.0030 (7)
C4	0.0282 (10)	0.0225 (10)	0.0185 (9)	−0.0023 (8)	0.0119 (8)	−0.0019 (7)
C5	0.0301 (11)	0.0271 (10)	0.0219 (10)	0.0060 (8)	0.0102 (8)	0.0001 (8)
C6	0.0220 (10)	0.0371 (12)	0.0287 (11)	−0.0007 (9)	0.0064 (9)	−0.0081 (9)
C7	0.0290 (11)	0.0275 (11)	0.0393 (12)	−0.0081 (9)	0.0140 (10)	−0.0062 (9)
C8	0.0301 (11)	0.0197 (10)	0.0283 (11)	−0.0014 (8)	0.0138 (9)	−0.0005 (8)
C9	0.0283 (11)	0.0274 (11)	0.0263 (10)	−0.0026 (8)	0.0116 (9)	0.0046 (8)
C10	0.0200 (10)	0.0245 (10)	0.0232 (10)	−0.0038 (7)	0.0105 (8)	0.0019 (8)
C11	0.0233 (10)	0.0377 (12)	0.0260 (11)	−0.0018 (8)	0.0128 (9)	−0.0017 (9)
C12	0.0253 (11)	0.0591 (15)	0.0216 (10)	−0.0011 (10)	0.0097 (9)	0.0073 (10)
C13	0.0219 (11)	0.0422 (13)	0.0402 (13)	0.0006 (9)	0.0051 (10)	0.0176 (10)
C14	0.0222 (11)	0.0292 (11)	0.0481 (14)	0.0001 (8)	0.0073 (10)	−0.0029 (10)
C15	0.0246 (11)	0.0318 (11)	0.0299 (11)	−0.0029 (8)	0.0090 (9)	−0.0037 (9)

### Benzylammonium phenylacetate (1) Geometric parameters (Å, º)

**Table:**

O1—C1	1.250 (2)	C7—C8	1.388 (3)
O2—C1	1.272 (2)	C7—H7	0.9500
N1—C9	1.494 (2)	C8—H8	0.9500
N1—H11	0.97 (2)	C9—C10	1.501 (3)
N1—H12	1.01 (2)	C9—H9A	0.9900
N1—H13	0.95 (3)	C9—H9B	0.9900
C1—C2	1.525 (2)	C10—C11	1.385 (3)
C2—C3	1.511 (2)	C10—C15	1.392 (3)
C2—H2A	0.9900	C11—C12	1.385 (3)
C2—H2B	0.9900	C11—H11A	0.9500
C3—C8	1.388 (3)	C12—C13	1.378 (3)
C3—C4	1.396 (2)	C12—H12A	0.9500
C4—C5	1.386 (3)	C13—C14	1.381 (3)
C4—H4	0.9500	C13—H13A	0.9500
C5—C6	1.384 (3)	C14—C15	1.385 (3)
C5—H5	0.9500	C14—H14	0.9500
C6—C7	1.379 (3)	C15—H15	0.9500
C6—H6	0.9500		
			
C9—N1—H11	109.1 (13)	C8—C7—H7	119.8
C9—N1—H12	110.4 (11)	C7—C8—C3	120.88 (18)
H11—N1—H12	109.2 (17)	C7—C8—H8	119.6
C9—N1—H13	109.0 (14)	C3—C8—H8	119.6
H11—N1—H13	111.2 (19)	N1—C9—C10	110.87 (15)
H12—N1—H13	107.9 (17)	N1—C9—H9A	109.5
O1—C1—O2	124.09 (15)	C10—C9—H9A	109.5
O1—C1—C2	118.04 (15)	N1—C9—H9B	109.5
O2—C1—C2	117.86 (15)	C10—C9—H9B	109.5
C3—C2—C1	113.80 (14)	H9A—C9—H9B	108.1
C3—C2—H2A	108.8	C11—C10—C15	118.89 (17)
C1—C2—H2A	108.8	C11—C10—C9	120.98 (17)
C3—C2—H2B	108.8	C15—C10—C9	120.05 (17)
C1—C2—H2B	108.8	C12—C11—C10	120.66 (19)
H2A—C2—H2B	107.7	C12—C11—H11A	119.7
C8—C3—C4	118.27 (17)	C10—C11—H11A	119.7
C8—C3—C2	120.20 (16)	C13—C12—C11	120.06 (19)
C4—C3—C2	121.53 (16)	C13—C12—H12A	120.0
C5—C4—C3	120.69 (17)	C11—C12—H12A	120.0
C5—C4—H4	119.7	C12—C13—C14	119.87 (19)
C3—C4—H4	119.7	C12—C13—H13A	120.1
C6—C5—C4	120.34 (18)	C14—C13—H13A	120.1
C6—C5—H5	119.8	C13—C14—C15	120.2 (2)
C4—C5—H5	119.8	C13—C14—H14	119.9
C7—C6—C5	119.42 (18)	C15—C14—H14	119.9
C7—C6—H6	120.3	C14—C15—C10	120.26 (19)
C5—C6—H6	120.3	C14—C15—H15	119.9
C6—C7—C8	120.39 (18)	C10—C15—H15	119.9
C6—C7—H7	119.8		
			
O1—C1—C2—C3	59.1 (2)	C2—C3—C8—C7	179.60 (17)
O2—C1—C2—C3	−122.12 (17)	N1—C9—C10—C11	−86.5 (2)
C1—C2—C3—C8	110.26 (18)	N1—C9—C10—C15	90.2 (2)
C1—C2—C3—C4	−70.2 (2)	C15—C10—C11—C12	−1.6 (3)
C8—C3—C4—C5	0.3 (2)	C9—C10—C11—C12	175.07 (17)
C2—C3—C4—C5	−179.29 (16)	C10—C11—C12—C13	0.4 (3)
C3—C4—C5—C6	−0.3 (3)	C11—C12—C13—C14	1.0 (3)
C4—C5—C6—C7	−0.1 (3)	C12—C13—C14—C15	−1.3 (3)
C5—C6—C7—C8	0.4 (3)	C13—C14—C15—C10	0.0 (3)
C6—C7—C8—C3	−0.4 (3)	C11—C10—C15—C14	1.4 (3)
C4—C3—C8—C7	0.0 (3)	C9—C10—C15—C14	−175.34 (18)

### Benzylammonium phenylacetate (1) Hydrogen-bond geometry (Å, º)

*Cg*1 is the centroid of the C3–C8 ring.

**Table:**

*D*—H···*A*	*D*—H	H···*A*	*D*···*A*	*D*—H···*A*
N1—H11···O1^i^	0.97 (2)	1.77 (2)	2.7177 (19)	165 (2)
N1—H12···O2^ii^	1.01 (2)	1.73 (2)	2.7306 (19)	170.1 (18)
N1—H13···O2^iii^	0.95 (3)	1.85 (3)	2.7938 (19)	174 (2)
C2—H2*B*···O1^iv^	0.99	2.40	3.375 (2)	169
C8—H8···O1^iii^	0.95	2.63	3.539 (2)	161
C9—H9···*Cg*1^v^	0.95	2.92	3.877 (2)	163

Symmetry codes: (i) −*x*+1/2, *y*+3/2, −*z*+1/2; (ii) −*x*+1/2, *y*+1/2, −*z*+1/2; (iii) *x*, *y*+1, *z*; (iv) −*x*+1/2, −*y*−1/2, −*z*+1; (v) *x*, −*y*+1, *z*−1/2.

### Benzylammonium phenylacetate (2) Crystal data

**Table:**

C_7_H_10_N^+^·C_8_H_7_O_2_^−^·H_2_O	*F*(000) = 560
*M**_r_* = 261.31	*D*_x_ = 1.227 Mg m^−^^3^
Monoclinic, *P*2_1_/*n*	Mo *K*α radiation, λ = 0.71073 Å
*a* = 6.8235 (7) Å	Cell parameters from 4619 reflections
*b* = 7.8766 (7) Å	θ = 2.7–25.0°
*c* = 26.364 (2) Å	µ = 0.09 mm^−^^1^
β = 93.218 (3)°	*T* = 100 K
*V* = 1414.7 (2) Å^3^	Block, colourless
*Z* = 4	0.10 × 0.06 × 0.04 mm

### Benzylammonium phenylacetate (2) Data collection

**Table:**

Bruker D8 Venture TXS diffractometer	2461 independent reflections
Radiation source: rotating anode (TXS), Bruker TXS	2090 reflections with *I* > 2σ(*I*)
Focusing mirrors monochromator	*R*_int_ = 0.043
Detector resolution: 7.4074 pixels mm^-1^	θ_max_ = 25.0°, θ_min_ = 3.5°
mix of phi and ω scans	*h* = −7→7
Absorption correction: multi-scan (SADABS; Bruker, 2016)	*k* = −9→8
*T*_min_ = 0.814, *T*_max_ = 0.971	*l* = −31→27
7319 measured reflections	

### Benzylammonium phenylacetate (2) Refinement

**Table:**

Refinement on *F*^2^	0 restraints
Least-squares matrix: full	Hydrogen site location: mixed
*R*[*F*^2^ > 2σ(*F*^2^)] = 0.075	H atoms treated by a mixture of independent and constrained refinement
*wR*(*F*^2^) = 0.170	*w* = 1/[σ^2^(*F*_o_^2^) + 3.8665*P*] where *P* = (*F*_o_^2^ + 2*F*_c_^2^)/3
*S* = 1.25	(Δ/σ)_max_ < 0.001
2461 reflections	Δρ_max_ = 0.30 e Å^−^^3^
187 parameters	Δρ_min_ = −0.27 e Å^−^^3^

### Benzylammonium phenylacetate (2) Special details

**Table:**

Geometry. All esds (except the esd in the dihedral angle between two l.s. planes) are estimated using the full covariance matrix. The cell esds are taken into account individually in the estimation of esds in distances, angles and torsion angles; correlations between esds in cell parameters are only used when they are defined by crystal symmetry. An approximate (isotropic) treatment of cell esds is used for estimating esds involving l.s. planes.
Refinement. C-H constr N-H and O-H: refxyz

### Benzylammonium phenylacetate (2) Fractional atomic coordinates and isotropic or equivalent isotropic displacement parameters (Å^2^)

**Table:**

	*x*	*y*	*z*	*U*_iso_*/*U*_eq_	
O1	0.2350 (3)	0.5687 (3)	0.47484 (9)	0.0213 (6)	
O2	0.4238 (4)	0.7897 (3)	0.45801 (10)	0.0245 (6)	
O3	0.2403 (4)	0.0283 (3)	0.51346 (10)	0.0230 (6)	
H32	0.296 (6)	−0.048 (6)	0.4981 (15)	0.028*	
H31	0.355 (6)	0.089 (5)	0.5279 (15)	0.028*	
N1	0.0153 (5)	0.2727 (4)	0.45885 (12)	0.0205 (7)	
H11	0.091 (6)	0.364 (5)	0.4595 (14)	0.025*	
H12	−0.084 (6)	0.296 (5)	0.4786 (15)	0.025*	
H13	0.101 (6)	0.191 (5)	0.4742 (14)	0.025*	
C1	0.3709 (5)	0.6394 (4)	0.45153 (13)	0.0174 (7)	
C2	0.4705 (5)	0.5289 (5)	0.41280 (13)	0.0213 (8)	
H2A	0.5366	0.4332	0.4311	0.026*	
H2B	0.3676	0.4802	0.3892	0.026*	
C3	0.6199 (5)	0.6182 (4)	0.38174 (13)	0.0190 (8)	
C4	0.8017 (5)	0.6658 (4)	0.40418 (14)	0.0210 (8)	
H4	0.8333	0.6385	0.4388	0.025*	
C5	0.9377 (5)	0.7532 (5)	0.37627 (14)	0.0236 (8)	
H5	1.0604	0.7860	0.3921	0.028*	
C6	0.8942 (6)	0.7920 (5)	0.32577 (14)	0.0272 (9)	
H6	0.9862	0.8522	0.3069	0.033*	
C7	0.7154 (6)	0.7424 (5)	0.30294 (14)	0.0280 (9)	
H7	0.6851	0.7674	0.2681	0.034*	
C8	0.5802 (5)	0.6560 (5)	0.33093 (14)	0.0239 (8)	
H8	0.4583	0.6223	0.3149	0.029*	
C9	−0.0635 (5)	0.2271 (5)	0.40676 (13)	0.0226 (8)	
H9A	−0.1605	0.3136	0.3947	0.027*	
H9B	−0.1320	0.1165	0.4080	0.027*	
C10	0.0974 (5)	0.2156 (4)	0.36952 (13)	0.0198 (8)	
C11	0.0829 (6)	0.3063 (5)	0.32460 (14)	0.0253 (8)	
H11A	−0.0272	0.3778	0.3174	0.030*	
C12	0.2285 (6)	0.2938 (5)	0.28976 (14)	0.0330 (10)	
H12A	0.2175	0.3567	0.2590	0.040*	
C13	0.3896 (6)	0.1892 (5)	0.30013 (15)	0.0315 (10)	
H13A	0.4887	0.1800	0.2764	0.038*	
C14	0.4056 (6)	0.0986 (5)	0.34496 (14)	0.0273 (9)	
H14	0.5164	0.0278	0.3521	0.033*	
C15	0.2608 (5)	0.1105 (4)	0.37956 (14)	0.0222 (8)	
H15	0.2722	0.0472	0.4102	0.027*	

### Benzylammonium phenylacetate (2) Atomic displacement parameters (Å^2^)

**Table:**

	*U*^11^	*U*^22^	*U*^33^	*U*^12^	*U*^13^	*U*^23^
O1	0.0211 (13)	0.0190 (13)	0.0243 (13)	−0.0042 (10)	0.0064 (10)	−0.0027 (11)
O2	0.0237 (14)	0.0181 (13)	0.0321 (15)	−0.0035 (11)	0.0068 (10)	−0.0066 (12)
O3	0.0200 (14)	0.0212 (14)	0.0279 (15)	−0.0001 (11)	0.0032 (10)	−0.0061 (12)
N1	0.0205 (16)	0.0160 (16)	0.0254 (17)	−0.0024 (13)	0.0050 (13)	−0.0044 (14)
C1	0.0122 (17)	0.0197 (18)	0.0201 (18)	−0.0014 (14)	−0.0011 (13)	−0.0014 (15)
C2	0.0248 (19)	0.0182 (18)	0.0213 (19)	−0.0018 (15)	0.0051 (14)	−0.0035 (15)
C3	0.0200 (18)	0.0145 (17)	0.0229 (19)	0.0030 (14)	0.0052 (14)	−0.0045 (15)
C4	0.0241 (19)	0.0192 (18)	0.0197 (18)	−0.0003 (15)	0.0020 (14)	−0.0012 (15)
C5	0.0208 (18)	0.0188 (19)	0.031 (2)	0.0001 (15)	0.0034 (15)	−0.0036 (16)
C6	0.029 (2)	0.025 (2)	0.029 (2)	−0.0042 (17)	0.0114 (16)	−0.0020 (17)
C7	0.032 (2)	0.031 (2)	0.0213 (19)	0.0023 (18)	0.0045 (15)	0.0034 (17)
C8	0.0175 (18)	0.030 (2)	0.024 (2)	−0.0029 (16)	0.0018 (14)	−0.0048 (17)
C9	0.0228 (19)	0.0222 (19)	0.0229 (19)	−0.0023 (15)	0.0019 (14)	−0.0030 (16)
C10	0.0242 (19)	0.0157 (17)	0.0195 (18)	−0.0086 (15)	0.0026 (14)	−0.0055 (15)
C11	0.030 (2)	0.025 (2)	0.0211 (19)	−0.0030 (16)	−0.0035 (15)	−0.0024 (16)
C12	0.046 (3)	0.035 (2)	0.0176 (19)	−0.011 (2)	0.0033 (17)	0.0000 (18)
C13	0.029 (2)	0.039 (2)	0.028 (2)	−0.0050 (18)	0.0110 (16)	−0.0072 (19)
C14	0.028 (2)	0.025 (2)	0.029 (2)	−0.0025 (16)	0.0054 (16)	−0.0052 (17)
C15	0.029 (2)	0.0142 (17)	0.0236 (19)	−0.0006 (15)	0.0046 (15)	−0.0017 (15)

### Benzylammonium phenylacetate (2) Geometric parameters (Å, º)

**Table:**

O1—C1	1.270 (4)	C6—H6	0.9500
O2—C1	1.247 (4)	C7—C8	1.391 (5)
O3—H32	0.83 (4)	C7—H7	0.9500
O3—H31	0.97 (4)	C8—H8	0.9500
N1—C9	1.490 (4)	C9—C10	1.516 (5)
N1—H11	0.89 (4)	C9—H9A	0.9900
N1—H12	0.90 (4)	C9—H9B	0.9900
N1—H13	0.94 (4)	C10—C11	1.382 (5)
C1—C2	1.529 (5)	C10—C15	1.402 (5)
C2—C3	1.516 (5)	C11—C12	1.394 (5)
C2—H2A	0.9900	C11—H11A	0.9500
C2—H2B	0.9900	C12—C13	1.389 (6)
C3—C8	1.384 (5)	C12—H12A	0.9500
C3—C4	1.395 (5)	C13—C14	1.380 (6)
C4—C5	1.398 (5)	C13—H13A	0.9500
C4—H4	0.9500	C14—C15	1.385 (5)
C5—C6	1.382 (5)	C14—H14	0.9500
C5—H5	0.9500	C15—H15	0.9500
C6—C7	1.386 (5)		
			
H32—O3—H31	100 (4)	C6—C7—H7	120.0
C9—N1—H11	113 (3)	C8—C7—H7	120.0
C9—N1—H12	110 (2)	C3—C8—C7	121.3 (3)
H11—N1—H12	106 (4)	C3—C8—H8	119.4
C9—N1—H13	114 (2)	C7—C8—H8	119.4
H11—N1—H13	102 (3)	N1—C9—C10	112.0 (3)
H12—N1—H13	111 (3)	N1—C9—H9A	109.2
O2—C1—O1	124.3 (3)	C10—C9—H9A	109.2
O2—C1—C2	119.7 (3)	N1—C9—H9B	109.2
O1—C1—C2	116.0 (3)	C10—C9—H9B	109.2
C3—C2—C1	115.7 (3)	H9A—C9—H9B	107.9
C3—C2—H2A	108.3	C11—C10—C15	119.0 (3)
C1—C2—H2A	108.3	C11—C10—C9	120.3 (3)
C3—C2—H2B	108.3	C15—C10—C9	120.7 (3)
C1—C2—H2B	108.3	C10—C11—C12	120.6 (4)
H2A—C2—H2B	107.4	C10—C11—H11A	119.7
C8—C3—C4	118.3 (3)	C12—C11—H11A	119.7
C8—C3—C2	121.5 (3)	C13—C12—C11	119.9 (4)
C4—C3—C2	120.3 (3)	C13—C12—H12A	120.1
C3—C4—C5	120.7 (3)	C11—C12—H12A	120.1
C3—C4—H4	119.7	C14—C13—C12	119.9 (4)
C5—C4—H4	119.7	C14—C13—H13A	120.0
C6—C5—C4	120.2 (3)	C12—C13—H13A	120.0
C6—C5—H5	119.9	C13—C14—C15	120.3 (4)
C4—C5—H5	119.9	C13—C14—H14	119.9
C5—C6—C7	119.5 (3)	C15—C14—H14	119.9
C5—C6—H6	120.3	C14—C15—C10	120.3 (3)
C7—C6—H6	120.3	C14—C15—H15	119.8
C6—C7—C8	120.1 (3)	C10—C15—H15	119.8
			
O2—C1—C2—C3	−4.5 (5)	C6—C7—C8—C3	0.2 (6)
O1—C1—C2—C3	174.7 (3)	N1—C9—C10—C11	−126.9 (4)
C1—C2—C3—C8	−107.2 (4)	N1—C9—C10—C15	54.4 (4)
C1—C2—C3—C4	72.2 (4)	C15—C10—C11—C12	0.0 (5)
C8—C3—C4—C5	1.6 (5)	C9—C10—C11—C12	−178.8 (3)
C2—C3—C4—C5	−177.8 (3)	C10—C11—C12—C13	0.1 (6)
C3—C4—C5—C6	−0.7 (5)	C11—C12—C13—C14	−0.3 (6)
C4—C5—C6—C7	−0.5 (6)	C12—C13—C14—C15	0.5 (6)
C5—C6—C7—C8	0.8 (6)	C13—C14—C15—C10	−0.5 (6)
C4—C3—C8—C7	−1.4 (5)	C11—C10—C15—C14	0.2 (5)
C2—C3—C8—C7	178.1 (3)	C9—C10—C15—C14	178.9 (3)

### Benzylammonium phenylacetate (2) Hydrogen-bond geometry (Å, º)

**Table:**

*D*—H···*A*	*D*—H	H···*A*	*D*···*A*	*D*—H···*A*
O3—H32···O2^i^	0.83 (4)	1.90 (4)	2.728 (4)	175 (4)
O3—H31···O2^ii^	0.97 (4)	1.81 (4)	2.771 (4)	169 (3)
N1—H11···O1	0.89 (4)	1.92 (4)	2.791 (4)	168 (4)
N1—H12···O1^iii^	0.90 (4)	1.96 (4)	2.805 (4)	157 (4)
N1—H13···O3	0.94 (4)	1.87 (4)	2.809 (4)	170 (3)
C9—H9*B*···O3^iv^	0.99	2.51	3.196 (4)	126
C15—H15···O2^i^	0.95	2.57	3.412 (4)	147

Symmetry codes: (i) *x*, *y*−1, *z*; (ii) −*x*+1, −*y*+1, −*z*+1; (iii) −*x*, −*y*+1, −*z*+1; (iv) −*x*, −*y*, −*z*+1.
